# GLUT1 is a highly efficient L-fucose transporter

**DOI:** 10.1016/j.jbc.2022.102738

**Published:** 2022-11-22

**Authors:** Bobby G. Ng, Paulina Sosicka, Zhijie Xia, Hudson H. Freeze

**Affiliations:** Human Genetics Program, Sanford Burnham Prebys Medical Discovery Institute, La Jolla, California, USA

**Keywords:** L-fucose, D-glucose, membrane transporter, glycosylation, lectin, AAL, *Aleuria aurantia* lectin

## Abstract

Understanding L-fucose metabolism is important because it is used as a therapy for several congenital disorders of glycosylation. Exogenous L-fucose can be activated and incorporated directly into multiple N- and O-glycans *via* the fucose salvage/recycling pathway. However, unlike for other monosaccharides, no mammalian L-fucose transporter has been identified. Here, we functionally screened nearly 140 annotated transporters and identified GLUT1 (*SLC2A1*) as an L-fucose transporter. We confirmed this assignment using multiple approaches to alter GLUT1 function, including chemical inhibition, siRNA knockdown, and gene KO. Collectively, all methods demonstrate that GLUT1 contributes significantly to L-fucose uptake and its utilization at low micromolar levels. Surprisingly, millimolar levels of D-glucose do not compete with L-fucose uptake. We also show macropinocytosis, but not other endocytic pathways, can contribute to L-fucose uptake and utilization. In conclusion, we determined that GLUT1 functions as the previously missing transporter component in mammalian L-fucose metabolism.

Solute Carrier (SLC) proteins include nearly 460 transmembrane proteins among 65 families that enable the transit of small molecules across the plasma–lysosomal, endoplasmic reticulum, and Golgi membranes (1, 2, 3). Within this group, the SLC2A and SLC5A families of transporters are known to carry several neutral monosaccharides including glucose, galactose, mannose, glucosamine, fructose, and xylose (1, 4). The existence of human genetic disorders due to pathogenic mutations in several of these transporters highlights their importance in human biology (5). Perhaps the most widely studied of these monosaccharide transporters is *SLC2A1*, encoding GLUT1, which is widely expressed in many cells and tissue types, but plays an especially critical role in blood–brain barrier D-glucose transport (6). Mutations in *SLC2A1* cause severe infantile or less severe child-onset autosomal dominant neurological disorders, GLUT1 deficiency syndrome 1, [OMIM 606777] and GLUT1 deficiency syndrome 2, respectively [OMIM 612126] (7).

Unlike D-glucose, membrane transport of L-fucose in humans has not garnered interest despite several studies showing the clinical benefits of oral L-fucose in treating leukocyte adhesion deficiency type II [OMIM 266265], GFUS-CDG, and certain cancers (8, 9). Cellular fucosylation in mammals can be achieved *via* two mechanisms, *de novo* synthesis or salvage/recycling of L-fucose. The *de novo* pathway utilizes glucose as a substrate to generate GDP-fucose and requires two enzymes, GMDS and GFUS, whereas the salvage/(recycled) incorporates fucose directly into GDP-fucose and requires FCSK and FPGT (10) (Fig. 1*A*).

**Figure 1:**
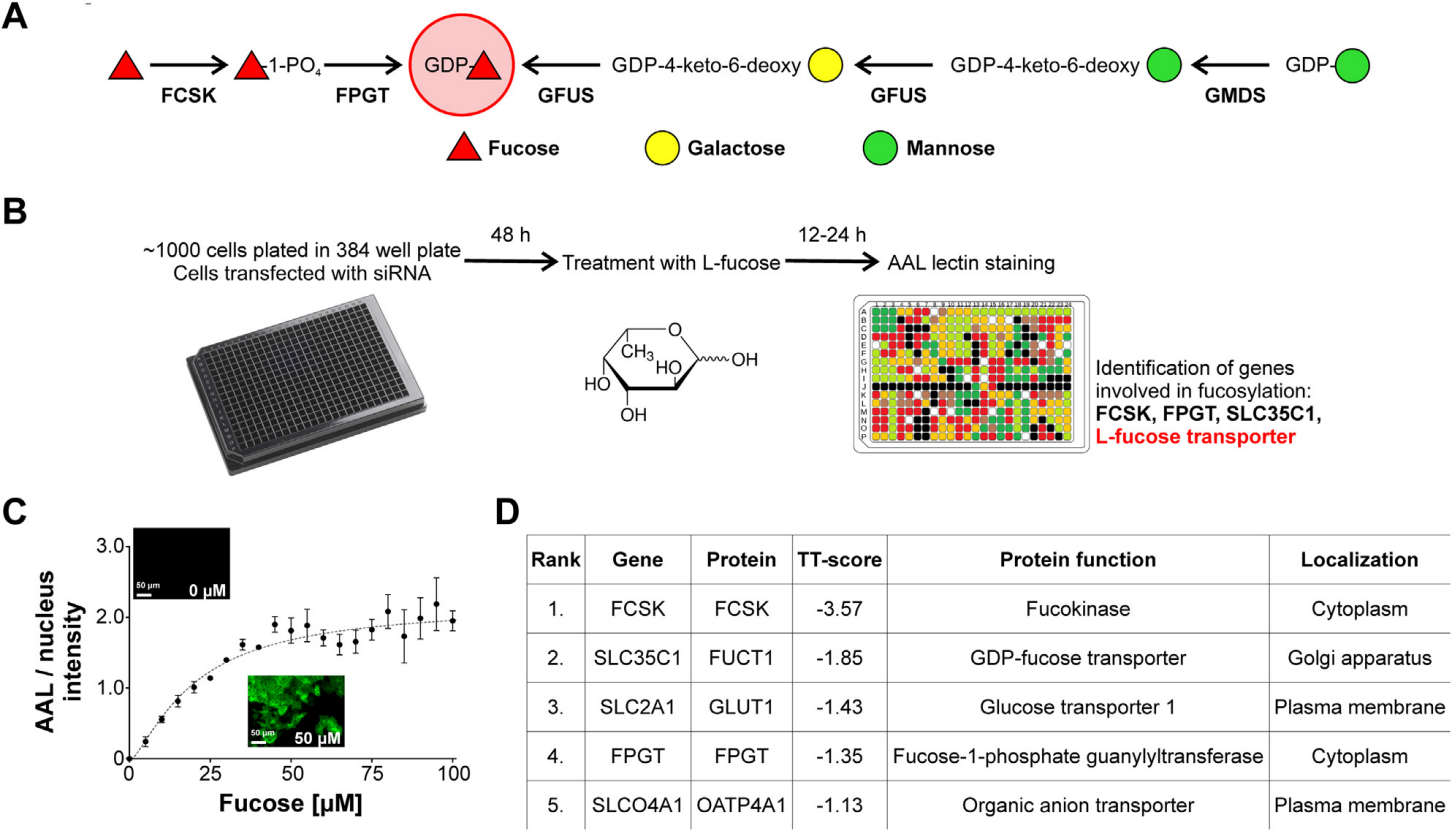
**Lectin-based assay identifies GLUT1 as plasma membrane L-fucose transporter.***A*, schematic showing both *de novo* and salvage fucosylation pathways. *B*, schematics of siRNA lectin assay workflow. *C*, saturation of AAL staining in increasing concentrations of exogenous L-fucose. Each point represents mean from three independent measurements ± SD. *D*, top hits identified during the siRNA assay exhibiting decreased AAL binding. TT-Score (Two-Target) is the ratio between lectin fluorescence signal and nuclei staining. FCSK, SLC35C1, and FPGT were used as controls. AAL, *Aleuria aurantia* lectin.

Since exogenous L-fucose is a therapeutic, we sought to identify an L-fucose transporter by developing a cell-based fluorescence assay using HCT116 colorectal cancer cells, which are deficient in the *de novo* GDP-fucose synthesis enzyme GMDS (11). These cells utilize an L-fucose salvage pathway that requires exogenous L-fucose to produce fucosylated N- and O-glycans (12). We screened an siRNA library of 140 SLC family members, including the Major Facilitator Superfamily of transporters known to transport other sugars, in order to identify candidates affecting the ability of L-fucose to enable fucosylation in HCT116 cells. Surprisingly, we found that GLUT1 significantly contributes to transport of L-fucose at low micromolar levels but mM levels of D-glucose do not compete. We show that loss of GLUT1 *via* chemical inhibitors, siRNA knockdown, and complete gene KO all result in similar loss of L-fucose uptake. In addition, we found that macropinocytosis and another, yet to be identified transporter, provide exogenous L-fucose, as well.

## Results

### siRNA screen identifies GLUT1 as an L-fucose transporter

Since HCT116 cells lack a functional *de novo* pathway, fucosylation is dependent on exogenous L-fucose. We used these cells to screen an siRNA library of approximately 140 genes (140 transporters and three positive controls) to identify a potential L-fucose transporter (Fig. 1*B*). We used the fucose-specific lectin, *Aleuria aurantia* lectin (AAL), which recognizes α1,2, α1,3, α1,4 (antennae), and α1,6 (core) linked fucose to visualize fucosylation. After optimizing fucose concentration and time (20 μM for 16 h), we performed the screen at 24 and 48 h post siRNA transfection (Fig. 1*C*). Importantly, siRNA knockdown of three positive controls *FCSK*, *FPGT*, and *SLC35C1* all showed a significant reduction in AAL reactivity (Fig. 1*D*). This reduction in these positive controls was used to establish our threshold for potential candidates. Only two candidates had comparable reduction in AAL reactivity; GLUT1 was prioritized since it is a well-established, verified monosaccharide transporter (Fig. 1*D*). The other candidate from our screen was *SLCO4A1*, which is annotated to encode for a Na^+^-independent transporter of organic anions (Fig. 1*D*). While it is unlikely to transport L-fucose, perhaps it transports molecules generated by fucose degradation such as fuconic acid (13).

### Chemical inhibition of GLUT1 function blocks L-fucose uptake

**Figure 2:**
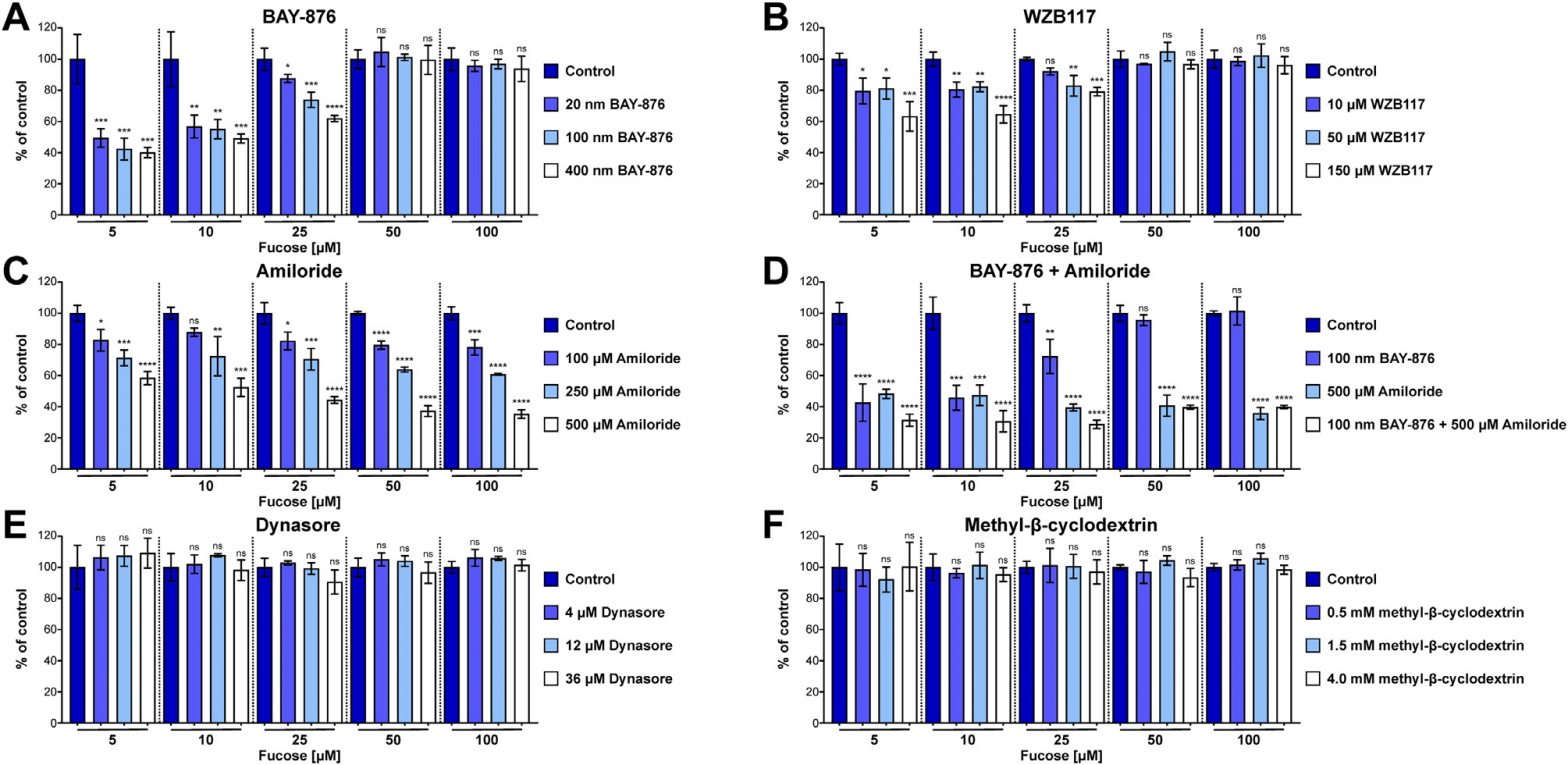
**Lectin-based assay measurements of L-fucose uptake in presence of GLUT1 and endocytosis inhibitors.***A*, L-fucose uptake in the presence of the GLUT1 inhibitor BAY-876 at increasing concentrations. *B*, L-fucose uptake in the presence of the GLUT1 inhibitor WZB117 at increasing concentrations. *C*, L-fucose uptake in the presence of the macropinocytosis inhibitor amiloride at increasing concentrations. *D*, L-fucose uptake in the presence of the BAY-876 and amiloride at increasing concentrations. *E*, L-fucose uptake in the presence of the endocytosis inhibitor Dynasore at increasing concentrations. *F*, L-fucose uptake in the presence of the endocytosis inhibitor methyl-β-cyclodextrin at increasing concentrations. One-way ANOVA with Bonferroni correction was used to assess statistical significance (ns > 0.05; ∗ ≤ 0.05; ∗∗ ≤ 0.01; ∗∗∗ ≤ 0.001; ∗∗∗∗ ≤ 0.0001). Each bar represents mean from three independent measurements ± SD.

BAY-876 is a potent, highly selective, cell-permeable GLUT1 inhibitor. At 20 nM, it reduced D-glucose uptake by 70%, while also reducing L-Fucose uptake by ∼50% (Fig. S1*A*) (Fig. 2*A*). Increasing the concentration of BAY-876 from 20 nM to 100 nM or 400 nM did not provide a significant reduction in L-fucose uptake but completely blocked D-glucose uptake (Fig. 2*A*) (Fig. S1*A*). This effect is comparable to the GLUT1 pooled siRNA used in the screen. We also tested the effects of another GLUT1 inhibitor, WZB117, which was not as potent as BAY-876 but showed a similar inhibitory trend (Fig. 2*B*). Interestingly, the inhibitory effect of BAY-876 could be overcome by increasing the concentration of exogenous L-fucose above 25 μM, suggesting a GLUT1-independent mode of entry at higher L-fucose concentrations (Fig. 2*A*).

### Inhibition of macropinocytosis affects L-fucose uptake

**Figure 3:**
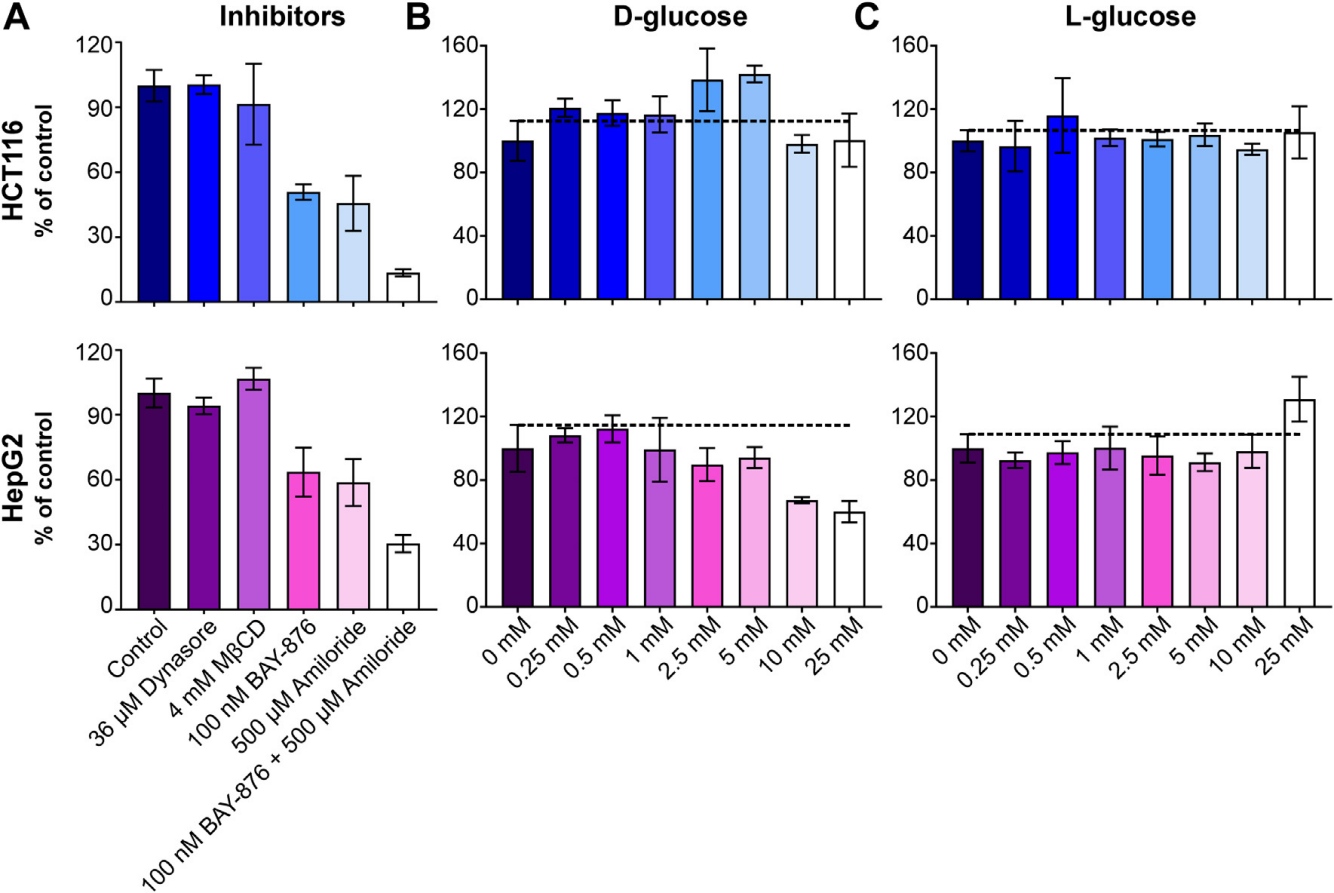
**Radioactivity-based measurement of L-fucose uptake.***A*, ^3^H-L-fucose uptake in the presence of clathrin (36 μM Dynasore) and caveolin (4 mM MBCD)-dependent endocytosis inhibitors, GLUT1 inhibitor (100 μM BAY-876) and macropinocytosis inhibitor (500 μM amiloride). *B*, ^3^H-L-fucose uptake using HCT116 (upper) or HepG2 (lower) cells in a presence of increasing concentrations of D-glucose. *C*, ^3^H-L-fucose uptake using HCT116 (upper) or HepG2 (lower) cells in a presence of increasing concentrations of L-glucose. Each bar represents mean from three independent measurements ± SD. MBCD, methyl-β-cyclodextrin.

We hypothesized that at higher L-fucose concentrations, macropinocytosis was a possible explanation for this affect, rather than being solely dependent on a transporter (14). This mechanism had been convincingly shown for the uptake and incorporation of Neu5Gc, a form of sialic acid (15). Using our high-throughput AAL detection method in HCT116 cells, we show inhibition of macropinocytosis with a nontoxic concentration (≤500 μM) of amiloride was effective in reducing L-fucose uptake in a dose-dependent manner and the combination of BAY-876 with amiloride was additive (Fig. 2, *C* and *D*). The combined treatment still permitted some L-fucose uptake, which could suggest another mechanism of entry. However, it should also be noted that amiloride can also act to affect sodium transport and we cannot completely exclude off target effects of this compound. Neither dynasore, which inhibits clathrin-dependent endocytosis, nor methyl-β-cyclodextrin, which inhibits caveolin-dependent endocytosis, affected L-fucose uptake in either the lectin-based assay or ^3^H-L-fucose uptake (Fig. 2, *E* and *F*) (Fig. 3*A*).

### D-glucose does not affect L-fucose uptake

In HCT116 cells, increasing concentrations of D-glucose even to hyperphysiological levels had a negligible effect on ^3^H-L-fucose uptake (Fig. 3*B* upper panel), suggesting that GLUT1-dependent D-glucose transport does not compete with L-fucose uptake. However, in HepG2 cells, which have a functional *de novo* biosynthetic pathway, there was an affect at higher D-glucose concentrations, but this is likely due to nonphysiological glucose flux through the fucose *de novo* pathway diluting of ^3^H-L-fucose–GDP pools with unlabeled glucose-derived GDP-fucose (Fig. 3*B* lower panel). As expected, L-glucose, which is not utilized by mammalian cells, has no effect and serves as a control (Fig. 3*C* upper and lower panel).

### GLUT1 KO cells have diminished L-fucose uptake

**Figure 4:**
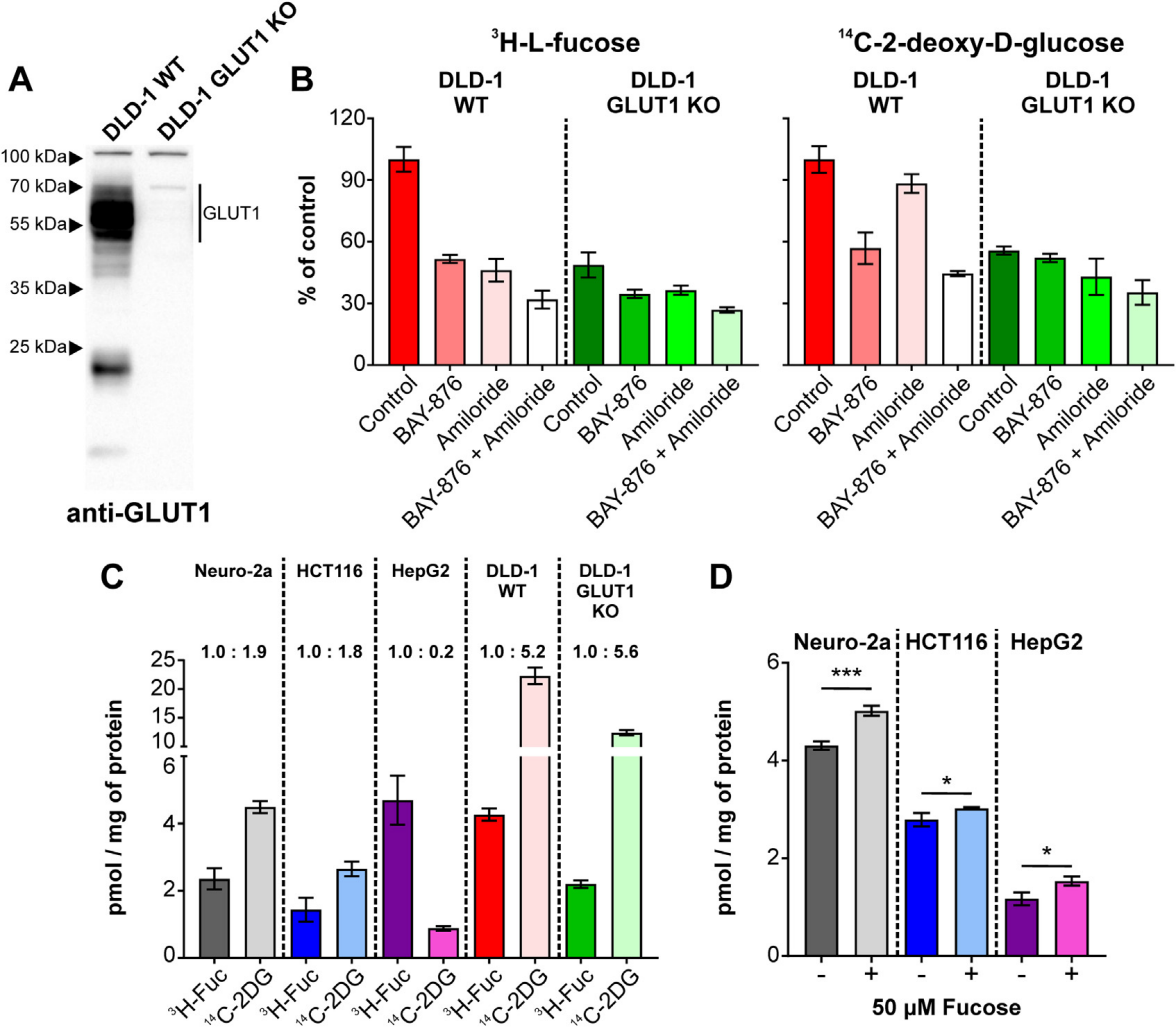
^**3**^**H-L-fucose and**^**14**^**C-2-deoxy-D-glucose uptake in DLD-1 WT and GLUT1 KO cells.***A*, Western Blot comparison of GLUT1 expression in DLD-1 WT and GLUT1 KO cells. *B*, comparison of three μM^3^H-L-fucose and ^14^C-2-deoxy-D-glucose uptake in DLD-1 WT and GLUT1 KO cells in a presence and absence of GLUT1 (100 μM BAY-876) and macropinocytosis inhibitors (500 μM Amiloride). *C*, amount of ^3^H-L-fucose and ^14^C-2-deoxy-D-glucose taken up by various cell lines. *D*, amount of ^14^C-2-deoxy-D-glucose taken up by various cell lines in a presence and absence of 50 μM fucose. Two-tailed, unpaired *t* test was used to assess statistical significance (ns > 0.05; ∗ ≤ 0.05; ∗∗ ≤ 0.01; ∗∗∗ ≤ 0.001; ∗∗∗∗ ≤ 0.0001). Each bar represents mean from three independent measurements ± SD.

While complete KO of GLUT1 is not compatible with life, genetically engineered KO cell lines do exist (16), as shown by the Western blots of DLD-1 WT and DLD-1 GLUT1 KO colorectal cancer cells (Fig. 4*A*). DLD-1 WT and DLD-1 GLUT1 KO colorectal cancer cells were simultaneously labeled with 3 μM each of ^3^H-L-fucose and ^14^C-2-deoxy-D-glucose to determine the contribution of GLUT1 to their uptake. As expected, when compared to WT cells, GLUT1 KO cells had reduced (∼50%), but not completely absent uptake of ^14^C-2-deoxy-D-glucose (Fig. 4*B* Right panel), proving there are other transporters for D-glucose. These same KO cells also showed a ∼60% reduction in ^3^H-L-fucose uptake compared to WT cells (Fig. 4*B* Left panel). We phenocopied these reductions on both ^14^C-2-deoxy-D-glucose and ^3^H-L-fucose by treating WT cells with BAY-876 (Fig. 4*B* Right and left panel). Amiloride had no effect on ^14^C-2-deoxy-D-glucose, while inhibiting ∼50% of ^3^H-L-fucose (Fig. 4*B* Right and left panel). Not surprisingly, treating GLUT1 KO cells with BAY-876 alone had a marginal effect on either ^14^C-2-deoxy-D-glucose or ^3^H-L-fucose uptake (Fig. 4*B* Right and left panel). However, there was still 40% to 50% uptake of both, supporting the existence of other, likely transporter-mediated, pathways.

### GLUT1 has preference for D-glucose over L-fucose

To determine whether GLUT1 prefers D-glucose or L-fucose transport at low micromolar levels levels, we simultaneously measured ^3^H-L-fucose and ^14^C-2-deoxy-D-glucose uptake (pmol/mg protein) in several cell lines. Under these conditions, GLUT1 shows 2- to 3-fold higher preference for glucose except in the hepatocellular carcinoma line HepG2, where the preference is reversed (Fig. 4*C*).

### Determination of human serum L-fucose concentrations

**Figure 5:**
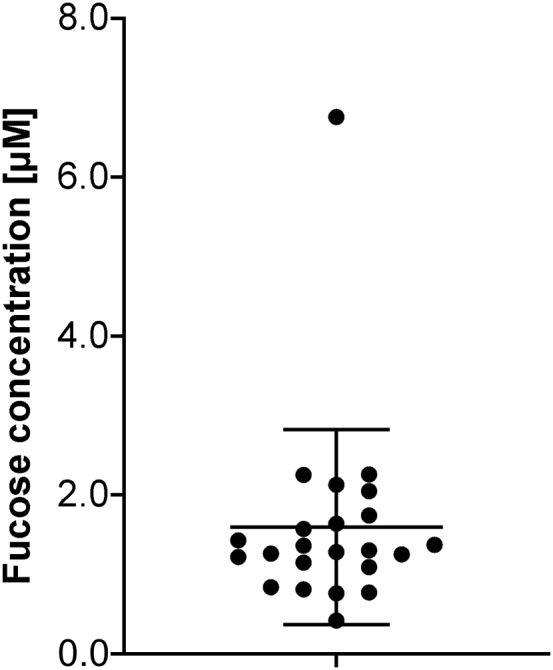
**GS-MS determination of L-fucose serum concentrations.** Serum concentration of free unbound L-fucose in 23 unrelated individuals. Data are presented as mean ± SD.

It has been shown oral L-fucose treatment with 50 to 100 mg/kg body weight can raise serum L-fucose levels to 110 to 210 μmol/l (17). Prior studies utilized coupled enzymatic assays to determine L-fucose concentrations in various conditions including cancer that in the healthy group ranged from 6 μM to over 500 μM (18, 19). Given the apparently low μM preference of GLUT1 for L-fucose, we next determined the serum concentrations for 23 unrelated individuals using a validated GC-MS method that allowed us to segregate L-fucose from other monosaccharides (12). We show that the average range is near 1.65 μM (Fig. 5)

## Discussion

The transport of monosaccharides provides the cell with energy and metabolic precursors used for a diverse range of processes. Understanding how these sugars are transported and utilized becomes especially important since various diseases/disorders (LADII, GFUS-CDG, SLC35A2-CDG, PGM1-CDG, colitis, various cancers) are already using or exploring “monosaccharide therapy” (9, 20, 21).

Previous work from the 1970s suggests exogenous L-fucose can only contribute ∼10% to total cellular fucosylation pools (22). However, this begs the question how such a small contribution could affect the phenotype of a specific disorder. More recent detailed dose-response experiments show this value is greatly underestimated and provides the first real clues to why L-fucose therapy is effective (12). Using GC-MS, we have determined free serum L-fucose concentrations to be ∼1.65 μM.

A putative L-fucose transporter of ∼57 kDa was purified from the mouse brain and was capable of transporting L-fucose in reconstituted liposomes with a *K*_*m*_ of ∼3 μM (23). This is nearly identical to the size of human GLUT1, which carries a single N-glycan. No further studies have been reported.

There are few sources of bioavailable L-fucose in the human diet. It is primarily used for glycoprotein synthesis, rather than energy production, although degradation pathways have been identified (24). While increasing exogenous L-fucose from 100 μM to >5 mM in hepatocellular carcinoma line, Hep3b, can increase cytosolic GDP-fucose levels, it has no effect on total cellular fucosylation (25). Varying exogenous L-fucose concentrations from 2 to 50 μM in multiple cell lines has no effect on the GDP-fucose concentration, showing that the total cytoplasmic pool is tightly regulated (12). However, increasing exogenous L-fucose increases its fractional contribution to glycosylation by suppressing the contribution of the *de novo* pathway (12). This study provided evidence that the cell can distinguish the origin of L-fucose and segregate those sources into multiple nonhomogenous pools of GDP-fucose for glycosylation. The mode of L-fucose entry (transporter *versus* micropinocytosis) might also influence its initial catabolism to fuconic acid, further degradation or secretion from the cell.

How GLUT1 transports L-fucose and D-glucose in a noncompetitive manner is both unknown and surprising. No previous GLUT1 studies examined L-fucose transport. Based on the measured rate of D-glucose uptake and its flux through GDP-mannose and eventually into glycoproteins, we estimate the maximal contribution of D-glucose for fucosylation is 1 in 50,000 molecules (26). That means that only 2 in 10^5^ transport events or GLUT1 molecules (or a combination of both) would provide enough fucose for full fucosylation, although this number is likely to be much lower.

It is plausible that a minor population of GLUT1 molecules distributes into membrane clusters and/or submicrodomains, including lipid rafts that affect its transport activity for L-fucose (27). It is also conceivable that an association of GLUT1 with other proteins or metabolism-dependent modification of GLUT1 itself could render it more suitable for transporting L-fucose. Compared to its D-glucose transport at physiological concentrations, only a tiny minority of GLUT1 molecules would be needed to deliver L-fucose for glycosylation.

Alternatively, we considered the possibility that a very rare GLUT1 conformation (1/50,000) exists, which in some way prefers L-fucose. Initial molecular modeling (R. Woods, unpublished observations) did not provide any obvious insights. Although, when aligning the known structures for GLUT1 and FucP (fucose transporter in *E.coli*), there was some suggestion for a potential L-fucose-binding site adjacent to the one for glucose. Further molecular dynamics modeling is a major commitment and was beyond the scope of this study. Further speculation is unwarranted at this time. In conclusion, the results of our study demonstrate that GLUT1 is a highly efficient L-fucose transporter.

## Experimental procedures

### Cell culture

HCT116, HepG2, and Neuro-2a cells (from ATCC) were grown in 1 g/l glucose Dulbecco’s modified Eagle’s medium (DMEM). DLD-1-WT and DLD-1-GLUT1-KO were maintained in 4.5 g/l glucose DMEM. All media was supplemented with 10% fetal bovine serum (Sigma-Lot 20J480) and cells grown at 37 °C in a humidified incubator at 5% CO_2_.

### Lectin staining of HCT116 cells

Lectin staining was performed as described by Sosicka *et al*. (12). Instead of biotinylated AAL, FITC-labeled AAL (Vector Laboratories), diluted 1:100, was used.

### siRNA screen

HCT116 cells were used to screen a panel of 143 siRNA (140 transports, three positive controls) and their effect on AAL staining. The initial screens used a pool of four combined siRNA with each candidate subsequently confirmed using individual siRNA. The lectin staining was performed essentially as described by Sosicka *et al*, except a 384-well format was used instead of 96-well.

### Inhibition of GLUT1, endocytosis, and macropinocytosis

GLUT1 was inhibited using 100 nM BAY-876 and 150 μM WZB117. Clathrin-dependent endocytosis was inhibited with 36 μM Dynasore. Caveolin-dependent endocytosis was inhibited with 4 mM methyl-β-cyclodextrin. Macropinocytosis was inhibited with 500 μM amiloride. All inhibitors were purchased from the Cayman Chemical. The toxicity of the inhibitors was assessed for each cell line. Prior to adding L-fucose, cells were preincubated with a respective inhibitor (or their combination) for 1 h.

### L-fucose uptake

L-fucose uptake was measured using L-fucose [5,6-^3^H] (60 Ci/mmol; American Radiolabeled Chemicals, Inc) that was QAE purified. Experiments were performed in serum-free DMEM 1 g/l glucose medium in 6-well plates. About 10 μCi/ml of radioactive fucose was used in each experiment. About 0.5 ml of the labeling medium was added into each well. Cells were grown in a presence of 5 μM unlabeled L-fucose (Sigma–Aldrich). L-fucose uptake was measured for 1 h. Before collecting cells, they were washed five times with Dulbecco's phosphate-buffered saline, scraped, and lysed in 2% SDS. Fifty percent of collected material was counted using a scintillation counter. Samples were normalized based on the protein concentration that was assessed with bicinchoninic acid assay method.

### L-fucose and 2-deoxy-D-glucose uptake

To measure an uptake of L-fucose and 2-deoxy-D-glucose at the same time, cells were grown in serum-free, glucose-free DMEM medium in 6-well plates. About 10 μCi/ml of QAE purified ^3^H-fucose and 0.8 μCi/ml of 2-deoxy-D-glucose [^14^C(U)] (274 mCi/mmol; Perkin Elemer) was used in each experiment. About 0.5 ml of the labeling medium was added into each well. Cells were grown in a presence of 3 μM unlabeled L-fucose, so the concentration of both fucose and 2-deoxyglucose was the same. Uptake was measured for 2 h. Before collecting cells, they were washed five times with DPBS, scraped, and lysed in 2% SDS. Fifty percent of collected material was counted using a scintillation counter. Samples were normalized based on the protein concentration that was assessed with bicinchoninic acid assay method.

### GC-MS analysis of serum fucose

To measure L-fucose serum concentration we spiked in 500 pmol of L-[UL-^13^C_6_] fucose (Omicron Biochemicals, Inc) to 100 μl of serum. To remove glycoproteins, we first added 400 μl of ice-cold acetone to each sample and incubated them for 16 h at −20 °C. Next, samples were spun down for 30 min, 4 °C, 14,000 rpm and resulting supernatant was dried in SpeedVac. Samples were dissolved in 200 μl of water, loaded on 3 kDa cutoff filters, and spun down for 15 min, 14,000 rpm, room temperature. Next, additional 200 μl of water was loaded on the filter and samples were spun down again. The flow through the filter was collected and dried in SpeedVac. Finally, the samples were derivatized and analyzed with GC-MS as previously described (12).

## Data availability

All data within the article will be shared upon request (corresponding author). Raw data for the GC-MS figure were deposited in Figshare https://figshare.com/projects/GCMS_files_for_Ng_et_al_GLUT1_is_a_Highly_Efficient_L-Fucose_Transporter_/150984.

## Supporting information

This article contains supporting information.

## Conflict of interest

The authors declare that they have no conflicts of interest with the contents of this article.
