# Supplementary figures and images for: GLUT1 is a highly efficient L-fucose transporter
Source: J Biol Chem. 2022 Nov 22;299(1):102738. doi: 10.1016/j.jbc.2022.102738 (PMC9758431; doi:10.1016/j.jbc.2022.102738)

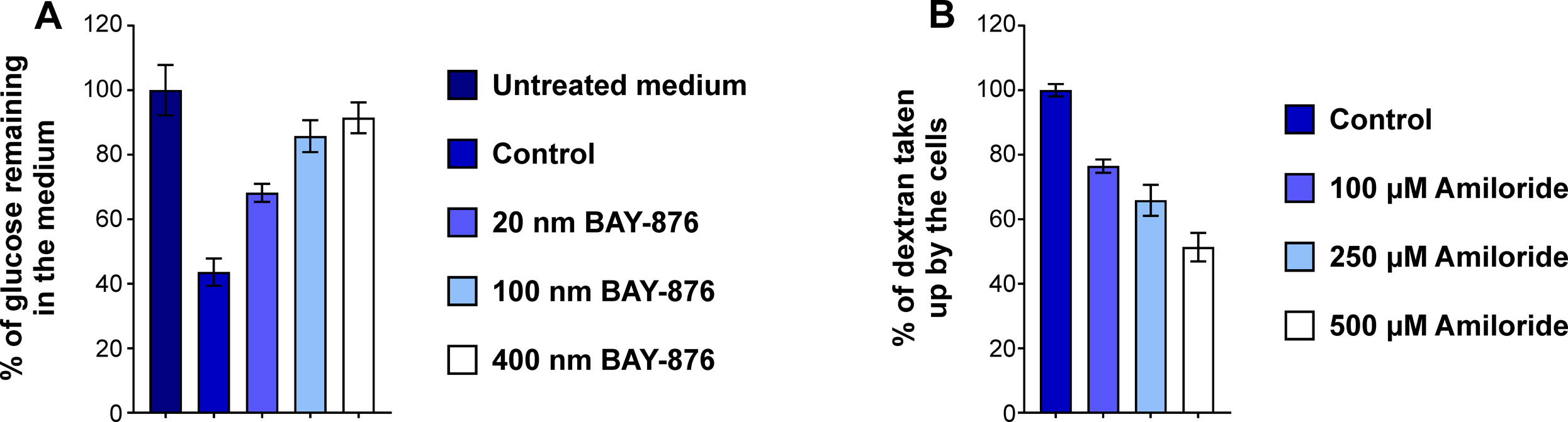

Supplement: Figure S1 [file figs1.jpg]
